# Supplementary material for: Quercivorol as a lure for the polyphagous and Kuroshio shot hole borers, Euwallacea spp. nr. fornicatus (Coleoptera: Scolytinae), vectors of Fusarium dieback
Source: PeerJ. 2017 Aug 17;5:e3656. doi: 10.7717/peerj.3656 (PMC5563438; doi:10.7717/peerj.3656)
Supplement: Supplemental Information 1 — Minimum, maximum, and mean values of number of shot hole borers captured per trap for each experiment. Standard error of the mean and Pearson’s dispersion statistic is also reported. [file peerj-05-3656-s001.docx]

Supplementary Materials

| **Experiment** | **Min** | **Max** | **Mean** | **SE** | **Pearson’s dispersion statistic** |
| --- | --- | --- | --- | --- | --- |
| Experiment 1 | 0 | 47 | 5.42 | 0.93 | 1.815765 |
| Experiment 2 | 0 | 232 | 24 | 2.05 | 8.96037 |
| Experiment 3 | 0 | 266 | 41.21 | 5.72 | 18.6935 |
| Experiment 4 | 2 | 661 | 89.27 | 11.72 | 75.4172 |
| Experiment 5 | 0 | 125 | 10.76 | 2.39 | 15.7298 |
| Experiment 6 | 0 | 310 | 44.83 | 5.52 | 21.6067 |
| Experiment 7 | 0 | 244 | 40.41 | 3.57 | 23.6081 |
| Experiment 8 | 0 | 216 | 30.24 | 1.94 | 22.3596 |
| Experiment 9 | 0 | 28 | 2.49 | 0.24 | 4.07333 |
| Experiment 10 | 0 | 169 | 14.26 | 2.31 | 7.38229 |
| Experiment 11 | 0 | 119 | 10.43 | 1.15 | 5.24621 |

**Table S1** Minimum, maximum, and mean values of number of shot hole borers captured per trap for each experiment. Standard error of the mean and Pearson’s dispersion statistic is also reported.
